# A New Paradigm for Uterine Fibroid Treatment: Transcervical, Intrauterine Sonography-Guided Radiofrequency Ablation of Uterine Fibroids with the Sonata System

**DOI:** 10.1007/s13669-017-0194-2

**Published:** 2017-02-15

**Authors:** David B. Toub

**Affiliations:** 1 Gynesonics, Inc, 301 Galveston Drive, Redwood City, CA 94063 USA; 2 Department of Obstetrics and Gynecology, Albert Einstein Medical Center, 5501 Old York Road, Philadelphia, PA 19141 USA

**Keywords:** Fibroids, Radiofrequency ablation, Sonata, Intrauterine sonography

## Abstract

**Purpose of Review:**

This article provides the current evidence related to transcervical radiofrequency ablation of uterine fibroids under integrated intrauterine sonography guidance (the Sonata System).

**Recent Findings:**

Published data on the treatment of fibroids with the Sonata System has demonstrated significant median reductions in total (73.3%) and perfused (73.3%) uterine fibroid volume, menstrual bleeding (72.3%), symptom severity (62.5%), and improvements in health-related quality of life (127%) at 12 months post-ablation. A clinical trial under an FDA Investigational Device Exemption is in progress.

**Summary:**

The Sonata System is a promising treatment modality for uterine fibroids. As an incisionless, minimally invasive treatment that does not require general anesthesia or hospitalization, it has the potential for redefining the current paradigm for management of symptomatic fibroids.

## Introduction

Uterine fibroids (leiomyomata uteri) are benign solid tumors that affect the majority of women in the USA by age 50 [1]. While often asymptomatic, fibroids can result in abnormal uterine bleeding, pelvic pressure, subfertility, dyspareunia, and other symptoms. Uterine fibroids are the leading indication for hysterectomy in the USA, Europe, and other countries [2–4]. While treatment options (hysterectomy, myomectomy, uterine artery embolization) exist, they typically involve major surgery and inpatient admission, require incisions and general anesthesia, and can be associated with significant adverse events and prolong the return to the activities of daily living. Hysteroscopic myomectomy is limited to smaller submucous fibroids, with some requiring staggered treatment with multiple partial resections to avoid inadvertent serosal injury along with fluid overload and other complications [5].

A 2013 study indicated that nearly 80% of women expressed an interest in minimally invasive options for fibroid treatment and 51% reported a desire to conserve their uteri; of particular note, 25% delayed treatment up to 5 years [6]. The situation is similar in Europe, as Downes and colleagues found that nearly 33% of Italian women with symptomatic fibroids, and almost a quarter of fibroid patients in the UK, waited over 5 years before obtaining treatment [4].

In response to the desire for novel, minimally invasive solutions for women with fibroids who desire uterine conservation, there has been a growing interest in the use of radiofrequency (RF) energy to ablate uterine leiomyomata. The successful application of RF ablation to solid tumors of the liver and other organs has affirmed the validity of this approach [7–9].

The Sonata® System (Gynesonics; Redwood City, CA), previously known as VizAblate™, integrates radiofrequency ablation for the treatment of fibroids with intrauterine sonography for real-time imaging within a single medical device [10]. It is CE-marked (Conformité Européene)in the European Union and is currentlyunder investigation in the USA. Because it is placed transcervically, it is incisionless and does not require general anesthesia. In particular, Sonata enables the outpatient treatment of a wide range of uterine fibroid types. While Sonata can treat FIGO (Fédération Internationale de Gynécologie et d'Obstétrique) type 1 and type 2 fibroids, it can also ablate fibroids that are not treatable with hysteroscopic methods (e.g., FIGO types 3, 4, 5, 6 and types 2–5 [transmural]).

This paper will provide an overview of transcervical radiofrequency ablation (RFA) for uterine fibroids, review the current evidence base for the Sonata System, and offer a perspective on its potential role in the armamentarium of general and specialized obstetrician-gynecologists who manage the many women with symptomatic uterine leiomyomata.

## Transcervical Radiofrequency Ablation of Uterine Fibroids

As a hyperthermic energy source, radiofrequency energy heats soft tissue to effect coagulative necrosis. The zone of coagulative necrosis undergoes granulation and other inflammatory responses, resulting in fibrosis and volume reduction.

While radiofrequency ablation of uterine fibroids dates to the early 1990s, these earlier efforts did not involve concurrent imaging in order to match the volume of ablated tissue to that of the targeted fibroid nor was the energy delivered transcervically [11–14]. The use of concurrent sonography has enabled a volumetric approach to RFA, which allows the operator to minimize the number of ablations necessary to ablate most or all of the targeted fibroid. And while earlier devices for radiofrequency ablation required either laparotomy or laparoscopy, sonographic guidance has enabled a transcervical approach, obviating the need for surgical incisions [15–20].

Unlike the situation with malignancies, it is not necessary to ablate 100% or more of a fibroid’s volume to provide sustained clinical benefit [21]. Nonetheless, it is preferable to ablate as much of a fibroid as is safely possible, as early clinical evidence suggests a higher efficacy with increasing percentages of ablated fibroid volume [21–23]. Furthermore, fibroids that have been sufficiently ablated are rendered largely or entirely necrotic, and while some tissue may remain in situ, patients can realize symptom relief. When thermal energy is delivered transcervically, it is important to ensure that heat is not transferred to the uterine serosa and beyond, as that may be adhesiogenic and risk thermal injury to adjacent organs such as the bowel and the bladder. Graphical guidance systems can be helpful, both to assist with fibroid targeting and to deliver energy within the serosal margin [24].

## Overview of the Sonata System

The Sonata System consists of a reusable intrauterine ultrasound (IUUS) probe and a single-use disposable RFA handpiece with proprietary Graphical Guidance Software (GGS) for diagnosis and targeting. These components are integrated to provide the gynecologist with a real-time image-guided treatment system.

The IUUS probe is used to identify fibroids from within the uterine cavity and guide deployment of an introducer and needle electrodes into one or more targeted fibroids. The intrauterine sonography image offers a unique high-resolution perspective of the uterus and nearby structures, including the bowel, the bladder, and the adnexae. The IUUS probe image is curvilinear, penetrates more than 9 cm, has a transmit frequency of 4.8–9.0 Mhz, and provides a 90° field of view. In conventional transvaginal sonography, the endometrial stripe is a useful landmark. But with intrauterine sonography, the IUUS probe resides within the endometrial cavity proper. The imaging plane is always directed at a 90° sector to the sonography probe, so there is a single imaging plane rather than the sagittal and coronal planes that make transvaginal sonography perhaps more challenging.

**Fig. 1:**
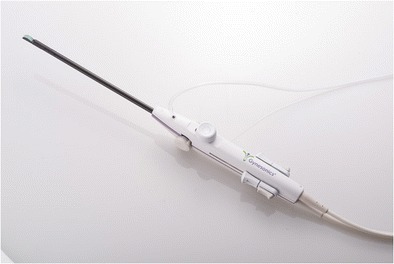
The Sonata treatment device (combination of IUUS probe and RFA handpiece)

The RFA handpiece is a single-use component that contains an introducer and needle electrode array. The RFA handpiece snaps together with the IUUS probe to form and integrate into a single treatment device (Fig. 1) that contains all the controls for the physician to place and size the ablation. Mechanical stops and lockouts within the RFA handpiece provide definitive Mechanical stops and lockouts within the RFA handpiece provide definitive physical endpoints, ensuring the ablation is properly located and sized as selected by the physician using the included graphical software.

The Sonata Graphical Guidance Software, also known as the SMART Guide™, is a real-time graphical overlay on the ultrasound display. The SMART Guide allows the operating gynecologist to visually select the deployment length, width, and position of the ablation guides (thus setting the mechanical stops for the introducer and needle electrodes) before any hardware has been inserted into the fibroid target. By displaying the ellipsoidal region where the ablation will take place (ablation zone), along with a surrounding ellipsoid (thermal safety border) where tissue temperatures will be elevated, the SMART Guide may provide a safer and more accurate fibroid ablation. These ellipsoidal guides were validated in more than 4000 ablations in bovine muscle and human extirpated uteri, both ex vivo, as well as in vivo during laparotomy (during early preclinical testing in which serosal temperatures were monitored with direct placement of thermocouples and concurrent infrared camera measurements of surface temperatures).

**Fig. 2:**
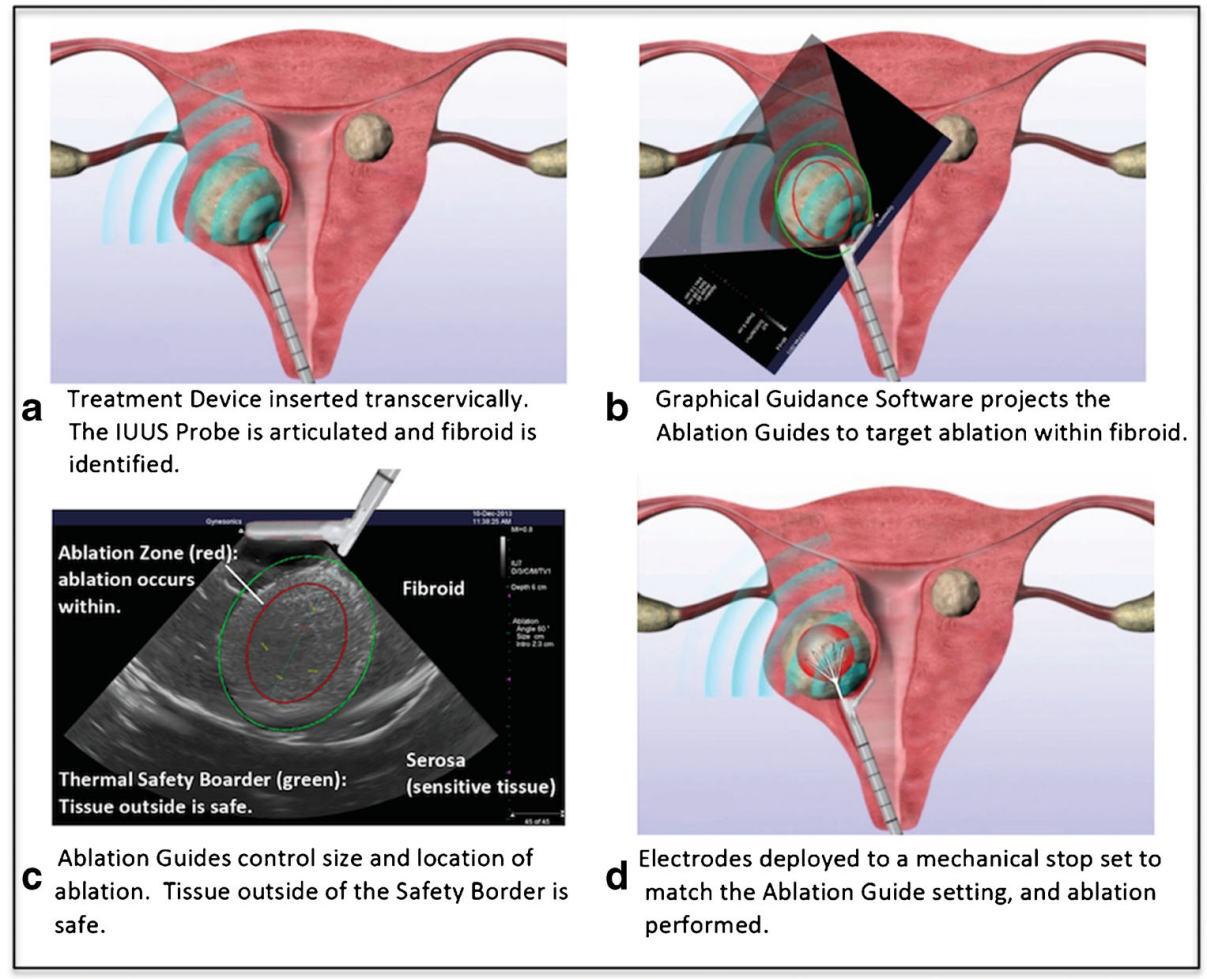
Transcervical RF ablation with the Sonata System, demonstrating the SMART Guide that delineates the ablation zone (red ellipsoid) and thermal safety border (green ellipsoid. Note explanation for each panel within the Figure)

Sonographically, the serosa appears as a hyperechoic structure. By using the SMART Guide, an ablation is positioned in order to encompass as much of the fibroid as possible while keeping thermal energy within the uterine serosal margin (Fig. 2). Once the desired ablation size is selected and safe placement of the needle electrodes is confirmed by rotating the IUUS probe in multiple planes, therapeutic RF energy is delivered to the fibroid according to a fixed treatment cycle that is dependent on ablation size. The system is designed to modulate power (up to 150 W) to keep temperatures at the needle electrode tips around 105 °C, and the time at temperature (2–7 min) is automatically set based on the ablation size that the gynecologist has selected via the SMART Guide. The Sonata System can create a continuous range of ablation sizes up to 4.0 cm wide and up to 5.0 cm long. Multiple ablations may be created within a single fibroid.

Unlike operative hysteroscopy or transcervical fibroid morcellation, only a small amount of hypotonic solution is instilled within the endometrial cavity. This is performed for acoustic coupling of the IUUS probe rather than for significant uterine distension. The diameter of the treatment device, which is comprised of both the IUUS probe and RFA handpiece attached to one another, is 8.3 mm, compatible with a cervical dilatation of 27 French.

The Sonata System, with its IUUS probe coupled to another device (in this case, a RFA handpiece), forms a platform in which other potential devices may be attached to the IUUS probe and provide additional functionality beyond ablation.

## Clinical Evidence

There is a growing evidence base relative to transcervical, intrauterine sonography-guided RFA of fibroids with the Sonata System.

## Ablation Data After Immediate and Delayed Hysterectomy

A single-site cohort study was performed involving 19 women (20 fibroids) undergoing either immediate (*n* = 12) or delayed (*n* = 7) hysterectomy after transcervical RF ablation with the VizAblate System (as Sonata was formerly known) [24]. The study was intended to demonstrate the ability of the device to ablate the majority of fibroid volume in fibroids up to 5.0 cm in diameter while avoiding the uterine serosa. Hysterectomies were performed via laparotomy in all cases, with the delayed cohort having their hysterectomies 2 weeks after transcervical RF fibroid ablation. All patients in the delayed hysterectomy group received conscious sedation during their transcervical ablation procedures, whereas those in the immediate hysterectomy group were managed with epidural anesthesia. Ablation percentages in ablated fibroids were ascertained after hysterectomy by staining the extirpated uteri with the viability stain triphenyltetrazolium chloride (TTC) to quantify fibroid ablation dimensions and assess the serosa for thermal injury. There were 17 fibroids ≤5.0 cm in diameter among the 19 patients, in which a median 75% of fibroid volume was ablated (mean 67.2 ± 27.0%; range 15–100%). No uteri were found on either gross visualization at hysterectomy or upon histopathologic evaluation to have evidence for uterine serosal injury.

## European Clinical Trial Data: the Fibroid Ablation Study-EU Trial

The Fibroid Ablation Study-EU (FAST-EU) Trial was a multicenter, prospective trial in the Netherlands, the UK, and Mexico, that examined the clinical effects of the VizAblate System in a cohort of women with symptomatic fibroids [15, 16]. Each patient served as her own control, and an independent core imaging laboratory was used to ensure quality control and standardized interpretation of baseline and post-ablation magnetic resonance images (MRI). Patients had to have up to five treatable fibroids from 1 to 5 cm in diameter, not desire future fertility, and were excluded for the presence of type 0 myomata, ovulatory dysfunction, coagulopathy, and adenomyosis. All patients had to have at least one fibroid that indented the endometrial cavity (FIGO types 1 and 2), a menstrual pictogram (MP) score of at least 120 and a baseline score of 20 or greater on the Symptom Severity Score (SSS) subscale of the Uterine Fibroid Symptom-Quality of Life (UFS-QOL) questionnaire. Anesthesia was per physician and patient choice.

The primary endpoint of the FAST-EU trial was the percentage change in perfused volume of ablated fibroids at 3 months as determined by contrast-enhanced MRI. Reduction in perfused fibroid volume is a representation of how much of the ablated fibroid is devascularized after ablation and is believed to correlate with treatment success and durability of symptom improvement [21]. Other endpoints, which were reached at 6 months, included reduction in the MP score (which represents the degree of menstrual blood loss), overall safety, improvements in the SSS and health-related quality of life (HRQOL) subscales of the UFS-QOL, patient satisfaction, anesthesia regimen, and recovery pain.

A total of 50 women (92 fibroids) were treated with transcervical RFA under intrauterine sonography guidance. All patients had undergone baseline transvaginal sonography, hysteroscopy, or hysterosonography, as well as contrast-enhanced MRI. Of the 92 fibroids treated, 56 (60.9%) were either a type 1 (*n* = 14) or a type 2 (*n* = 42) fibroid, which is consistent with the trial inclusion requirement for the presence of at least one indenting myoma. Ablated fibroid diameters, as determined by baseline MRI, ranged from 1.1–6.9 cm.

At 3 months, there was a 76.9% median reduction in perfused fibroid volume and a 62.5% median decrease in mean total fibroid volume (*P* < 0.001). By 12 months, among 28 patients who provided their consent, contrast-enhance MRI revealed a 73.3% median reduction in perfused fibroid volume and a 73.3% median reduction in total fibroid volume (*P* < 0.001).

With regard to menstrual bleeding, by 3 months, 89.8% of patients reported a reduction in menstrual bleeding, with the mean MP score decreasing consistently through 12 months (Table 1). Lukes and colleagues have determined that a reduction in menstrual bleeding ≥22% is considered to be clinically meaningful to women with abnormal uterine bleeding [25]. In the FAST-EU trial, most patients (57.1–72.9%, based on the time point) realized more than a 50% reduction in their MP scores during the study, while 75.5% of patients achieved a clinically meaningful reduction in their MP scores by 3 months. Similarly, there were significant improvements (all *P* < 0.001 from baseline) in the SSS and HRQOL subscales through 12 months (Table 1). A 10-point reduction in SSS is considered to represent a moderate effect size [26]. Patients in the FAST-EU trial realized a mean 35.3-point reduction in their SSS scores at 12 months, with as many as 86% of patients at a single time point achieving at least a 10-point reduction.

**Table 1:** Median percentage improvement in patient-reported outcomes from baseline

	3 months (%)	6 months (%)	12 months (%)
MP	56.9	68.6	72.3
SSS	52.5	66.7	62.5
HRQOL	123	118	127

*MP* menstrual pictogram, *SSS* Symptom Severity Score, *HRQOL* Health-related Quality of Life (the last two are subscales of the Uterine Fibroid Symptom-Quality of Life questionnaire)

Patients returned to normal activity in a median 4.0 days (mean 4.4 ± 3.1 days) and there was an overall satisfaction rate of 87.8%. Two patients were admitted overnight for observation, one with lower abdominal pain believed secondary to cystitis and the other for bradycardia after treatment received under general anesthesia. The most frequent adverse events consisted of dysmenorrhea (12%), abnormal uterine bleeding above baseline (12%), pelvic pain/cramping (8%), and cystitis (4%). One patient had a fibroid expulsion that was free of sequelae. Four patients underwent elective reintervention between 7 and 12 months post-ablation, two with hysteroscopic myomectomy, one with balloon thermal endometrial ablation, and one with total abdominal hysterectomy.

There was a single pregnancy, which was diagnosed 6 months after ablation when the patient presented with 3 months of amenorrhea [27]. This resulted in a live-born singleton pregnancy delivered by elective repeat Cesarean section at term, with an uncomplicated perinatal course.

## Future Research Efforts

The Sonata System is currently being evaluated as part of an FDA-approved investigational device exemption (IDE) trial (SONATA: Sonography-Guided Transcervical Ablation of Uterine Fibroids; ClinicalTrials.gov Identifier: NCT02228174) involving clinical sites in the USA, Mexico, and Europe [28]. This trial aims to show the safety and efficacy of transcervical RFA of uterine fibroids associated with heavy menstrual bleeding.

As noted, successful pregnancy after treatment with the Sonata System (formerly VizAblate) has been reported. There is a growing evidence base concerning the potential for women who have undergone hyperthermic ablation with either focused ultrasound, microwaves, or radiofrequency energy to experience normal fertility and fecundity [20, 27, 29–36]. Nonetheless, radiofrequency ablation of uterine fibroids in women who desire fertility remains investigational in the USA, although treatment in women desiring pregnancy has been approved by the US Food and Drug Administration in the case of MR-guided focused ultrasound (MRgFUS), another hyperthermic ablation modality. In order to examine whether transcervical RFA of uterine fibroids with the Sonata System conserves the structure of the myometrium, baseline and 12-month post-ablation MR images will be examined as part of the ongoing pivotal trial with regard to myometrial thickness and integrity. Additional clinical trials are planned to provide further evidence regarding the role, if any, of transcervical RF ablation in women who desire fecundity.

## Discussion and Perspective

While hysterectomy and other more invasive options remain prevalent, there remains a need for less invasive fibroid therapy that is incisionless, preserves the uterus, and can treat fibroids that are not limited to the narrow range of fibroid types (FIGO types 0 and 1 and smaller type 2 fibroids) amenable to transcervical treatment with a hysteroscope. It is established that hysteroscopic management of type 2 fibroids may be particularly challenging; hysteroscopic resection of type 2 fibroids may have a 50% probability of requiring at least one additional attempt at complete removal [37, 38].

The Sonata System, because it has an integrated intrauterine sonography probe, is not limited to submucous fibroids visible with hysteroscopic methods. The only fibroids that would not be ablated with Sonata are pedunculated, namely FIGO type 0 and type 7 myomata. Ablation of a type 7 fibroid (pedunculated subserous myoma) would require ablation outside of the uterine serosa. Type 0 (intracavitary/pedunculated submucous) myomata are generally amenable to hysteroscopic resection [39, 40]. Unlike uterine artery embolization of submucous myomata, which is more commonly associated with bulk fibroid expulsion, hyperthermic ablation technologies such as MRgFUS and RFA tend to result in a gradual sloughing of fibroid tissue from the endometrial cavity, with bulk expulsion being much less common [16, 39, 41].

Because the imaging and treatment components of the Sonata System are integrated into a single handheld device, the operator only manages one device and one image, rather than coordinate multiple devices (e.g., laparoscope, RF device, sonography probe) and images (video, sonography). Intrauterine sonography provides a different imaging perspective from that of transvaginal sonography. As the curvilinear IUUS probe always maintains the same relationship to the portion of the uterus being imaged (i.e., there are no separate coronal and sagittal planes depending on how the sonography probe is angled), the imaging plane remains consistent while scanning the entire circumference of the uterus. The uterine serosa is an important landmark, appearing as a hyperechoic border, and demarcates the limit beyond which the SMART Guide’s thermal safety border must not pass.

The unique perspective and high-resolution imaging afforded by the IUUS probe enables precise targeting and RF ablation of a wide range of fibroid types, while the SMART Guide provides a real-time visual display (ablation zone) of where the ablation will occur along with the constraint of the thermal safety border. Because the mechanical stops within the RFA handpiece limit needle electrode deployment based on the ablation size graphically chosen by the operator, no manual measurements are needed to ascertain where the needle electrodes should be deployed. The system automatically calculates the time at temperature (105 °C) based on the chosen ablation size and will stop the delivery of RF energy when the necessary time at temperature has elapsed (2–7 min).

Radiofrequency ablation of uterine fibroids with the Sonata System is a promising treatment approach that is currently investigational in the USA and available under CE mark in Europe. As a transcervically delivered fibroid treatment, the uterine serosa is not compromised. Unlike hysteroscopic resection, tissue is not morcellated nor is significant and prolonged uterine distention necessary. There is evidence in the literature for ablation of fibroids up to 6.9 cm with the Sonata System, and fibroids larger than 5.0 cm (the maximum deployment length of the needle electrodes) may be approached with multiple ablations [10, 16]. Neoadjuvant treatment with gonadotropin-releasing hormone agonists or selective progesterone receptor modulators, which medically reduce fibroid volume, may be a complementary approach to enable the optimal ablation of larger fibroids.

In an era in which physicians are increasingly performing more invasive hysterectomies and myomectomies in the wake of the US Food and Drug Administration guidance regarding laparoscopic power morcellation, it is important that women continue to have options for uterine fibroid therapy that are less invasive and preserve their choices with regard to uterine conservation [42]. The Sonata System, by virtue of being a transcervical procedure that has a wider range of treatable fibroid types than existing transcervical options, could significantly change the current paradigm in which women either lose their uteri or potentially undergo surgical and radiologic procedures that have significant drawbacks in terms of invasiveness and recovery time or require multiple treatment sessions.

## Conclusions

Because it is incisionless, does not require general anesthesia, can ablate most types of uterine fibroids, and preserves the uterus, the Sonata System represents an exciting new technology for the treatment of uterine fibroids. This has been supported to date by the results of the FAST-EU trial in Europe and Mexico, in which there were significant reductions in fibroid volume and fibroid-associated symptoms. The ongoing pivotal IDE trial (SONATA) will provide further evidence regarding clinical outcomes and safety in a mostly US-based patient cohort.
